# Modeling and Analysis of System Error for Highly Curved Freeform Surface Measurement by Noncontact Dual-Axis Rotary Scanning

**DOI:** 10.3390/s21020554

**Published:** 2021-01-14

**Authors:** Li Miao, Linlin Zhu, Changshuai Fang, Ning Yan, Xudong Yang, Xiaodong Zhang

**Affiliations:** State Key Laboratory of Precision Measuring Technology & Instruments, Laboratory of Micro/Nano Manufacturing Technology, Tianjin University, Tianjin 300072, China; miaoliii@tju.edu.cn (L.M.); l_linzhu@tju.edu.cn (L.Z.); cshfang@tju.edu.cn (C.F.); yanning@tju.edu.cn (N.Y.); yangxudong@tju.edu.cn (X.Y.)

**Keywords:** freeform surface, dual-axis rotary scanning, system calibration

## Abstract

Profile measurement is a key technical enabler in the manufacturing of highly curved freeform surfaces due to their complex geometrical shape. A current optical probe was used to measure nearly rotary freeform surfaces with the help of one rotation axis, because the probe needs to measure along the normal vector of the surface under the limitation of the numerical aperture (NA). This kind of measuring system generally has a high cost due to the high-precision, multi-axis platform. In this paper, we propose a low-cost, dual-axis rotation scanning method for a highly curved freeform surface with an arbitrary shape. The optical probe can scan the surface profile while always keeping consistent with the normal vector of the measuring points with the help of the double rotation axis. This method can adapt to the changes in curvature in any direction for a highly curved freeform surface. In addition, the proposed method provides a system error calibration technique for the rotation axis errors. This technique can be used to avoid the dependence of the measuring system on the high-precision platform. The three key system errors that affect the measurement accuracy such as installation error of the B-axis, A-axis, and XZ perpendicularity error are first analyzed through establishing an error model. Then, the real error values are obtained by the optimal calculation in the calibration process. Finally, the feasibility of the measurement method is verified by measuring one cone mirror and an F-theta mirror and comparing the results to those obtained using commercial equipment. The maximum measurable angle of the system is ±90°, the maximum measurable diameter is 100 mm, and the measurement accuracy of the system reaches the micron level in this paper.

## 1. Introduction

Optical freeform surfaces are widely used in space optics, projection optical systems [1,2,3], medical endoscope systems [4,5,6], and other fields because of their complex surfaces and multiple degrees of freedom. With the increasing demand for freeform surfaces in the aerospace industry, national defense and the military, precision instruments, and other modern cutting-edge technology fields, a variety of freeform surfaces with a high degree of curvature and steepness have been proposed, and the accuracy of freeform surface manufacturing in various fields has become higher [7,8]. However, due to the complex geometric characteristics of highly curved freeform surfaces, the traditional measurement methods are limited by the measurement range and numerical aperture (NA, the product of the half-angle of the objective’s collection cone and the index of refraction of the immersion medium) [9] of the probe, which cannot precisely measure the full surface topography of a highly curved freeform surface. As a result, profile measurement has become a key technical enabler in the manufacturing and application of such components [10,11].

Compared with the traditional contact coordinate measuring machines or similar equipment, the noncontact coordinate point scanning measurement method is more efficient and does not scratch the surface. The sensor in a noncontact rotary coordinate point scanning measurement system is equipped with a rotating axis, which makes the relative position of the optical sensor and the workpiece more flexible. This flexibility effectively solves the problem of the sensors being unable to measure beyond the angular tolerance. However, the extra motion associated with flexibility brings about some challenges to the measurement accuracy of the system, and some measures need to be taken to ensure accuracy. Typical commercial measuring instruments for noncontact rotary coordinate point scanning include Nanomefos from TNO (Deft, The Netherlands) [12,13], LuphoScan from Taylor Hobson [14], and UltraSurf from OptiPro [15,16]. The accuracy of Nanomefos can reach 30 nm, which is sufficient to realize the measurement of a large aspheric surface area. However, these systems are too complex and expensive to use in production lines. LuphoScan and UltraSurf adopt the relative rotation scanning mode for the probe and the workpiece. However, LuphoScan realizes the real-time decoupling of the error by measuring the error of the motion system with the help of multiple laser interferometers. UltraSurf does not provide real-time compensation for motion error, and the measurement accuracy is limited by the accuracy of the moving platform. In these measuring devices, the probe has only one degree of rotational freedom, so most of these devices can only measure symmetrical, rotating structural parts. All of the abovementioned measuring instruments rely on a high-precision shafting motion or feedback positioning with the help of a laser interferometer. These systems are complex and expensive, and the requirements for the external environment are very strict.

The core problem with rotary scanning measurement is that the measurement system’s error is greater, the rotary axis system’s calibration is difficult, and the motion accuracy of the system’s displacement table is poor, all of which result in the rotary scanning coordinate measurement system having low efficiency and low measurement accuracy. The error modeling of and compensation in scanning systems with multiple degrees of freedom have become the primary bases of this kind of measurement equipment. In recent years, based on the choice of a high-precision motion platform, researchers have done a lot of work on error modeling and calibration of rotary scanning measurement systems. In terms of system error modeling, Rahman uses the homogeneous transfer matrix to establish the comprehensive spatial error model of the machine tool, which considers the geometric error, thermal error, rotary axis error, and elastic deformation error of the machine tool [17,18]. To date, this method is relatively advanced for geometric error modeling of a machine in the published work [19]. The main methods for calibrating a rotation axis are the reverse method [20], the three-point method [21], and the multi-point method [22]. In the error modeling of a measurement system with multiple degrees of freedom, Du proposed a self-calibration technique for a five-axis, laser-optical measurement system based on a ball bar [23]. He used the parameter estimation method to obtain the system model’s parameters through the ball bar. The calibration technology enables the system to achieve a measurement accuracy of better than 5 microns. Zhang set up a noncontact scanning measurement system for a four-axis blade profile [24]. He established a multi-body mathematical model to calculate the measurement space coordinate transformation matrix and used a three-beam interferometer and a standard gauge block to verify the geometric error of the system. The measurement results were improved to some extent, but some measurement errors remained. In summary, the existing rotational scanning measurement systems have been shown to be able to carry out 3D scanning measurement with rotational symmetry, such as measurement of a spherical surface. However, a method for the measurement of a nonrotationally symmetric freeform surface has yet to be developed. The existing rotary scanning measurement method is still limited by key technologies such as system error calibration and compensation.

In this paper, we propose a noncontact dual-axis rotary coordinate scanning measurement method based on the confocal to solve the problem of highly curved optical freeform surface measurement. In order to ensure surface information was always captured within the sensing range, Cheng, F. et al. developed an adaptive surface tracing algorithm [25]. However, this method may not meet the angular tolerance of the probe at some measuring points. The innovation of the method in this paper is that two orthogonal rotating axes are equipped with sensors for space-relative pose scanning measurement. Compared with the existing rotary scanning method, the proposed method has more degrees of freedom, and theoretically can adapt to the changes in curvature in any direction on a highly curved freeform surface. In addition, the proposed method provides a system error calibration technique for the rotation axis errors. The key system errors that affect the measurement accuracy are first analyzed through establishing an error model. Then, the real error values are obtained by the optimal calculation in the calibration process. The advantages of this method are that it avoids the use of additional high-precision equipment to calibrate the system and solves system error problems conveniently and cheaply. Finally, the feasibility of the proposed measurement method is verified by measuring typical devices and comparing the results with those obtained using advanced commercial equipment.

## 2. The Measurement Method of the Large Curvature Freeform Surface

**Figure 1:**
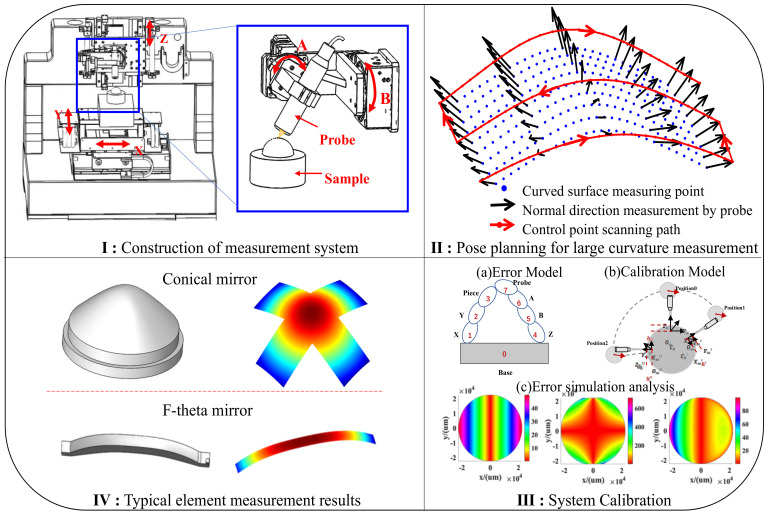
Measuring principle of the highly curved freeform surface measuring system.

As shown in Figure 1, a five-axis measurement system composed of three orthogonal axes (X, Y, Z) and two rotational axes (A, B) is first needed to construct the method for rotary scanning measurement of a highly curved freeform surface. Three translational axes are used to satisfy the basic motion of the system, and two rotational axes are mounted on the Z-axis to control the probe’s rotation in two dimensions to ensure that the probe can adapt to the changes in curvature in any direction on the highly curved freeform surface. During measurement, the position and the posture of the probe should be planned according to the profile of the measured surface, so that the probe can rotate along the normal vector of the measured surface to ensure that each measuring point falls within the measuring range and the NA value of the probe. However, we found that, during actual measurement, the system error seriously affects the measurement accuracy of the system, particularly the installation error of the rotating platform, which causes the actual measurement point to be unknown after the platform rotates and produces errors in the subsequent calculations. In this paper, we establish an error model for the entire measurement system, simulate and analyze the law and degree of influence of different errors, and obtain the key error terms that affect the measurement accuracy of the system. Then, the real error values are obtained by the optimal calculation in the calibration process. Finally, the feasibility of the measurement method proposed in this paper is proved by measuring two typical workpieces (a cone mirror and an F-theta mirror). The reliability of the measurement method proposed in this paper is proved by comparing it with those of commercial instruments.

## 3. Modeling and Simulation of Measurement System Error

### 3.1. Establishment of the Systematic Error Model

**Figure 2:**
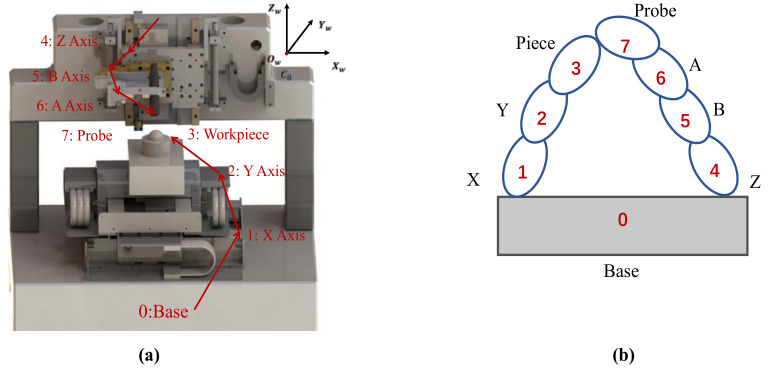
System model: (**a**) Structural model; (**b**) error model based on a multi-body mathematical model.

The structural model of the measurement system is shown in Figure 2a. The system has five degrees of freedom. The X and Y guides are fixed on the marble platform, the Z guide is vertically mounted on the gantry, and the rotating platforms A and B are mounted on the Z guide. The establishment of the global coordinate system of the measurement system is based on the guides in three directions. The X-axis, Y-axis, and Z-axis are parallel to the guides in three directions, respectively, and the positive direction complies with the rule of the right-hand coordinate system. The origin O of the coordinate system is the reference zero position of the probe when it is initially stationary, and the positive directions of the axes A and B are counterclockwise.

The error of the measurement system can be divided into static error and dynamic error according to the different mechanisms of influence. “Static error” refers to a series of errors caused by installation before the system moves, and “dynamic error” refers to the error caused by the motion of system components. According to the above classification, there are 45 geometric errors in the five-axis measurement system [26], as shown in Table 1, where *δ_mn_* stands for the translational error, *θ_mn_* stands for the angle error, *α_mn_* stands for the perpendicularity error, and *β_mn_* and *T_mn_* stand for the installation error of the rotation axis.

In this paper, a comprehensive error model of the measurement system is established by combining the actual measurement process with the multi-body error model shown in Figure 2b. In the modeling process, the low-order volume array method is used to describe the topological structure of the multi-body system. The homogeneous transfer matrix is used to represent the geometric characteristics of each part, and the attitude and position coordinates of the probe in the workpiece coordinate system are calculated, to establish the actual measurement motion and complete the measurement system error modeling. Taking the measuring system’s base as a zero-order volume, the topology includes two branches: One from the measurement system’s base, X-axis, and Y-axis to the workpiece; and the other from the base, Z-axis, B-axis, and A-axis to the probe. According to the topological structure of the measurement system, the geometric characteristics of each part are represented by a homogeneous characteristic matrix. The transformation matrix T between two adjacent bodies is divided into four parts: The ideal stationary eigenmatrix *^i^_j_T_p_*, the static error characteristic matrix *^i^_j_T_pe_*, the ideal motion characteristic matrix *^i^_j_T_s_*, and the motion error characteristic matrix *^i^_j_T_se_*. Then, the characteristic matrix between volume *i* and the adjacent low-order volume *j* is:

(1) Tji=TjipTjipeTjisTjise

The position coordinate of the measured point in the probe coordinate system *P_t_* is:

(2) $$P_{t}={\left[X_{t},Y_{t},Z_{t},1\right]}^{T}$$

The position coordinate of the measured point in the workpiece coordinate system *P_c_* is:

(3) $$P_{c}={\left[X_{c},Y_{c},Z_{c},1\right]}^{T}$$

The coordinates of the workpiece under the influence of two branches are the same, so:

(4) T03Pc=T07Pt

As the workpiece coordinate system coincides with the global coordinate system, the coordinates of the measured point in the global coordinate system can be calculated as follows:

(5) Pw=T03−1T07Pt

The coordinates of the actual measured point under the influence of different errors can be obtained through the above formula. The final surface error distribution can be obtained by analyzing the difference between the actual measured point and the ideal measured point.

### 3.2. Simulation and Analysis of Measurement System Error

In this section, the multi-body error model established in Section 3.1 is used to simulate the final measurement surface error caused by different types of errors. It is helpful for us to recognize the system error in advance, understand its influence range and law, identify the system error according to the measurement results, and finally guide the error compensation.

**Figure 3:**
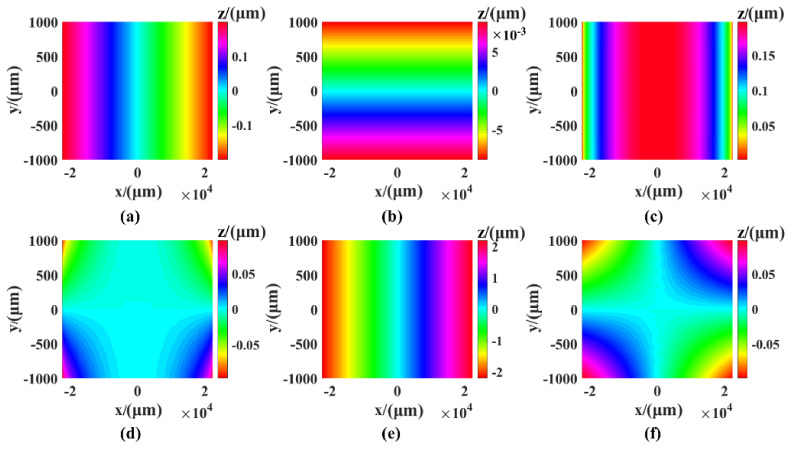
The shape error caused by X-axis movement: (**a**) Positioning error, (**b**) Y-direction straightness error, (**c**) Z-direction straightness error, (**d**) pitch angle error, (**e**) yaw angle error, (**f**) rolling angle error.

The dynamic error of the measuring system comes from the movement of the three linear axes and the two rotating axes. When a single linear axis moves, there are six degrees of freedom and six motion errors (a positioning error in the direction of motion, two straightness errors perpendicular to the direction of motion, and three deflection angle errors). We used the X-axis to simulate six motion errors, where the simulation range is X [–R, R], Y [–1 mm, 1 mm]. The dynamic error value is different from the static error value, which is fixed. Therefore, the system index was selected to be the simulation value during the simulation only for the error distribution range caused by our observations. The simulation values of the positioning error and the straightness error were 200 nm, and the simulation value of the three angle errors was 0.002°, which is close to the actual index. The simulation results are shown in Figure 3. It can be seen from the figure that the motion of the X-axis was not sensitive to the straightness error in the Y direction, the deflection angle error in the Y direction had the greatest influence on the surface shape error, and the peak valley value (PV) of error was 3.379 μm.

**Figure 4:**
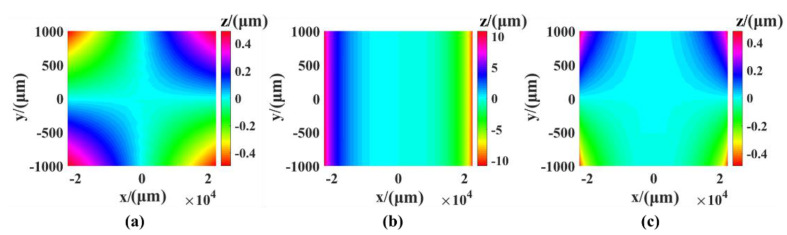
The shape error caused by the perpendicularity error: (**a**) XY perpendicularity error, (**b**) XZ perpendicularity error, (**c**) YZ perpendicularity error.

The static error of the measurement system has two main parts: The installation error of the rotating axis and the perpendicularity error between the linear axes. There are three linear guides in the measuring system, so there are three perpendicularity errors (the XY perpendicularity error, the XZ perpendicularity error, and the YZ perpendicularity error). The results of the simulation of the three errors are shown in Figure 4. The simulation value was 0.01°. It can be seen from the results that the surface shape error PV caused by the perpendicularity error between the X and Z axes reached 22.057 μm and had the greatest influence on the perpendicularity error PV of 0.98 μm between XY and YZ.

**Figure 5:**
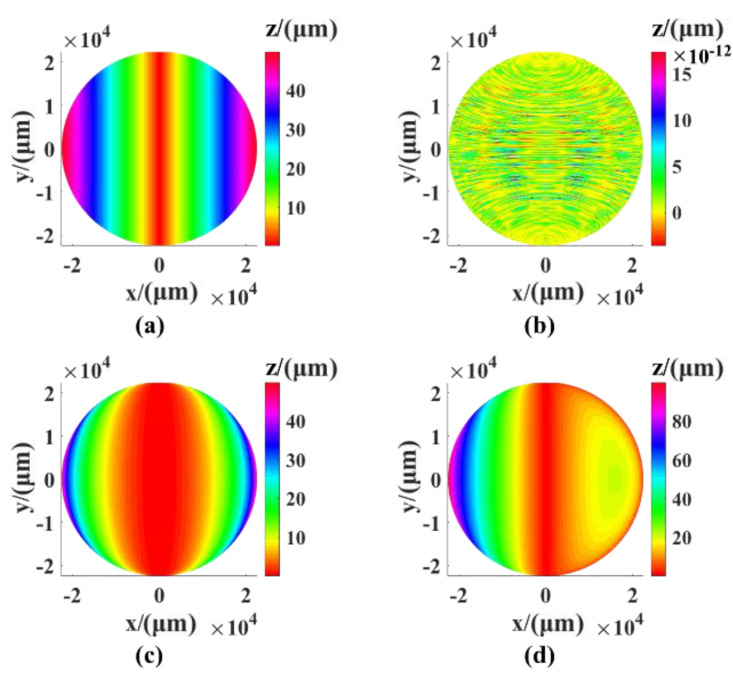
The shape error caused by the position deviation in different directions: (**a**) The X direction, (**b**) the Y direction, (**c**) the Z direction, and (**d**) the XYZ direction.

**Figure 6:**
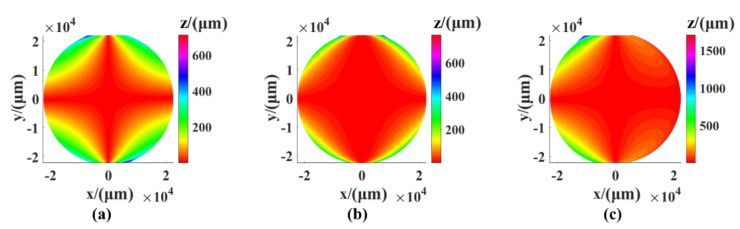
The shape error caused by the direction deviation in different directions: (**a**) The X direction, (**b**) the Z direction, and (**c**) the XZ direction.

The direction and pose of the rotation axis in the global coordinate system are unknown. Take rotation axis B as an example. Its ideal direction coincides with that of the Y-axis; however, it has components in the X and Z directions because of the installation error. Additionally, its position in the global coordinate system is also unknown, as the actual position and the ideal position have deviated in the X, Y, and Z directions, respectively. The results of the simulation of the error are shown in Figure 5 and Figure 6. It can be seen from the results that the position deviation of rotation axis B in the Y direction did not affect the measurement of the surface shape. However, when each deviation in the X direction and the Z direction was 50 μm, the position deviation of rotation axis B would cause a surface shape error of around 50 μm. The direction error of the rotation axis was larger than that of the position deviation, and the PV reached 1 mm.

### 3.3. Summary of Measurement System Error

The deviation between the ideal measured point and the actual measured point caused by the system error term can be obtained through the following system error model:

(6) $$E=P_{rel}-P_{ide}$$

where *P_rel_* is the real measured point and *P_ide_* is the ideal measured point. The influence of the error in *X*, *Y*, and *Z* directions can be expressed as follows:

(7) $$\begin{array}{l} E_{x}=-X*\cos\left(\partial_{\mathrm{yx}}\right)-\;X*\cos\left(B\right)-Z*\sin\left(B\right)-Y*\sin\left(\theta_{\mathrm{yz}}\right)+\cos\left(\partial_{\mathrm{yx}}\right)*Z*\sin\left(\partial_{\mathrm{zx}}\right)-\;Z*\left(\sin\left(\theta_{\mathrm{xy}}\right)\;+\;\sin\left(\theta_{\mathrm{yy}}\right)\right)+l(B_{0})\;\\ E_{y}=Z*\left(\sin\left(\theta_{\mathrm{xx}}\right)\;+\;\sin\left(\theta_{\mathrm{yx}}\right)\right)-\;T_{y}\;-\;Y*\cos\left(\partial_{\mathrm{yx}}\right)\;+\;X*\sin\left(\partial_{\mathrm{yx}}\right)\;-\;Z*\sin\left(\partial_{\mathrm{zy}}\right)\;+\;X*\sin\left(\theta_{\mathrm{xz}}\right)\;+\;X*\sin\left(\theta_{\mathrm{yz}}\right)+g(B_{0})\\ E_{z}=T_{z}+\;Z\;+\;Z*\cos\left(B\right)-\;X*\sin\left(B\right)\;-\;X*\sin\left(\theta_{\mathrm{xy}}\right)\;-\;X*\sin\left(\theta_{\mathrm{yy}}\right)\;+\;Y*\sin\left(\theta_{\mathrm{yx}}\right)+h(B_{0}) \end{array}$$

In Equation (7), the higher-order term of the error is ignored, because each error in the system is relatively small, and the higher-order term of the error is too small to calculate. After classifying and degenerating the errors, 13 errors were left (*θ_xx_*, *θ_xy_*, *θ_xz_*, *θ_yx_*, *θ_yy_*, *θ_yz_*, *α_zy_*, *α_xy_*, *α_zx_*, *Ty*, *T_z_*, *f*(*B*_0_), and *f*(*A*_0_)). The final measurement surface errors caused by these 13 errors were simulated, and the proportion of them to the total surface errors caused by these 13 errors was calculated. The results are shown in Table 2. It can be seen from the results that the errors of *α_zx_*, *f*(*B*_0_), and *f*(*A*_0_) had a great influence on the final shape error. Thus, the calibration of compensation for these three errors should be considered in the subsequent system calibration.

## 4. Calibration Model for the Measurement System

In Section 3 of this paper, the key errors of the system are determined and their effects on the final measurement results are analyzed, and Section 4 provides a method to quantify the system key error values determined in Section 3. From the analysis of system error in Section 3, we found that the installation errors *f*(*B*_0_) and *f*(*A*_0_) of the rotating axes and the XZ perpendicularity error had the greatest influence on the final surface shape measurement. Therefore, these errors were considered in the calibration model first. In this section, we establish a calibration model for the system and use this model to calibrate the actual installation pose of the rotating axis and the relationship between the X-axis and the Z-axis. We calculate the deviation between the true value and the ideal value, and compensate for it in the actual measurement process to accurately control the rotation and translation of the probe and achieve the goal of accurate measurement of the measured point.

**Figure 7:**
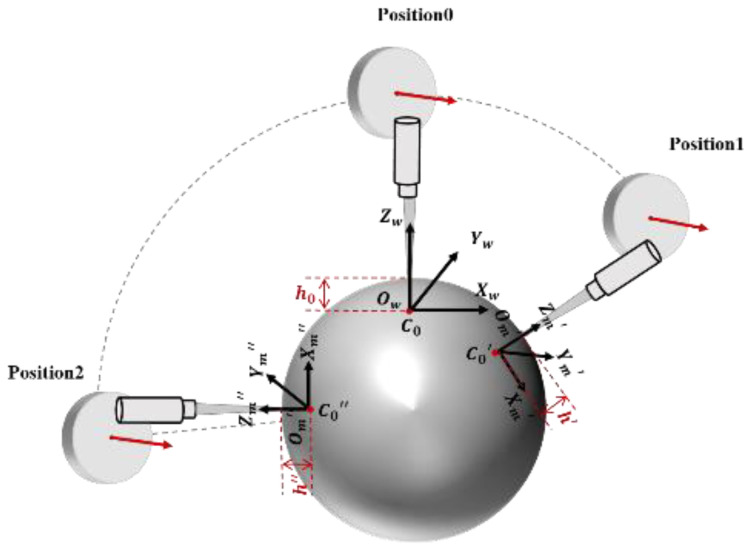
The system calibration model.

First, we discuss the installation position and pose of the rotating axis. We take a single rotating axis as an example, and the results can be compounded when there are two axes. The calibration model is shown in Figure 7. The direction and position of the central axis of the rotating axis are expressed by six unknown variables: The coordinates of any point of the central axis in the global coordinate system (*a*, *b*, *c*) and the vector of its direction (*l*, *m*, *n*). The reference zero position of the probe at the initial position is the origin of the global coordinate system: *C*_0_ (0, 0, 0). The indication of the probe *h*_0_ is the relative distance between the measured point and the reference zero point; thus, the coordinate of the measured point is *P*_0_ (0, 0, *h*_0_). The probe was rotated clockwise by θ degrees and translated by (*x*_1_, *y*_1_, *z*_1_) to Position 1. We established the measurement coordinate system with the reference zero position of the probe after rotation and translation as the origin O’M. The coordinates of the measured point in the measurement coordinate system are (0, 0, *h′*). The rotation transformation between the global coordinate system and the measurement coordinate system is represented by a matrix *M*. The coordinate of the measured point at Position 1 in the global coordinate system is:

(8) $$\left[\begin{array}{c} x^{\prime }\\ y^{\prime }\\ z^{\prime }\\ 1 \end{array}\right]=M\times \left[\begin{array}{c} 0\\ 0\\ h^{\prime }\\ 1 \end{array}\right]$$

The rotation transformation matrix for any point *P*_0_ that rotates *θ* degrees around the rotation axis determined by (*a*, *b*, *c*, *l*, *m*, *n*) is:

(9) $$\begin{array}{l} M=T\left(-a,-b,-c\right)\times R_{z}\left(-\gamma \right)\times R_{y}\left(-\beta \right)\times R_{x}\left(\theta \right)\times R_{y}\left(\beta \right)\times R_{z}\left(\gamma \right)\times T\left(a,b,c\right)\;\;\\ \;\;\;\;\;\;\;\;\;\;\;\;\;\;\;=\left[\begin{array}{l} l^{2}+\left(m^{2}+n^{2}\right)\cos\theta \;\;\;\;\;\;\;\;\;\;\;\;\;\mathrm{lm}\left(1-\cos\theta \right)-\mathrm{nsin}\theta \;\;\;\;\;\;\;\;\;\ln\left(1-\cos\theta \right)+\mathrm{msin}\theta \;\;\;\;\;\;\;\;\left(a\left(m^{2}+n^{2}\right)-l\left(\mathrm{bm}+\mathrm{cn}\right)\right)\left(1-\cos\theta \right)+\left(\mathrm{bn}-\mathrm{cm}\right)\sin\theta \\ \mathrm{lm}\left(1-\cos\theta \right)+\mathrm{nsin}\theta \;\;\;\;\;\;\;\;\;\;m^{2}+\left(l^{2}+n^{2}\right)\cos\theta \;\;\;\;\;\;\;\;\;\;\;\;\mathrm{mn}\left(1-\cos\theta \right)-\mathrm{lsin}\theta \;\;\;\;\;\;\;\;\;\;\;\;\;\;\left(b\left(l^{2}+n^{2}\right)-m\left(\mathrm{al}+\mathrm{cn}\right)\right)\left(1-\cos\theta \right)+\left(\mathrm{cl}-\mathrm{an}\right)\sin\theta \\ \ln\left(1-\cos\theta \right)-\mathrm{msin}\theta \;\;\;\;\;\;\;\;\;\;\mathrm{mn}\left(1-\cos\theta \right)+\mathrm{lsin}\theta \;\;\;\;\;\;\;\;\;\;n^{2}+\left(l^{2}+m^{2}\right)\cos\theta \;\;\;\;\;\;\;\;\;\;\;\;\left(c\left(l^{2}+m^{2}\right)-n\left(\mathrm{alm}+\mathrm{bm}\right)\right)\left(1-\cos\theta \right)+\left(\mathrm{am}-\mathrm{bl}\right)\sin\theta \\ \;\;\;\;\;\;\;\;\;\;\;\;\;\;\;\;0\;\;\;\;\;\;\;\;\;\;\;\;\;\;\;\;\;\;\;\;\;\;\;\;\;\;\;\;\;\;\;\;\;\;\;\;\;\;\;\;\;\;\;\;\;\;\;\;0\;\;\;\;\;\;\;\;\;\;\;\;\;\;\;\;\;\;\;\;\;\;\;\;\;\;\;\;\;\;\;\;\;\;\;\;\;\;\;\;\;\;\;\;\;\;\;\;0\;\;\;\;\;\;\;\;\;\;\;\;\;\;\;\;\;\;\;\;\;\;\;\;\;\;\;\;\;\;\;\;\;\;\;\;\;\;\;\;\;\;\;\;\;\;\;\;\;\;\;\;\;\;\;\;\;\;\;\;\;\;\;\;\;\;\;\;\;\;\;\;\;\;\;\;\;\;\;\;\;\;\;\;\;\;\;\;\;\;\;\;\;\;\;\;\;\;\;\;\;\;\;\;\;\;\;\;\;\;\;\;\;\;\;\;\;\;\;\;1 \end{array}\right] \end{array}$$

In this study, the standard ball was used as the calibration part, and the rotation axis was rotated by multiple angles sequentially to obtain the position *P*_1_, *P*_2_…*P_n_* of the corresponding rotation matrix *M*_1_, *M*_2_…*M_n_* in the global coordinate system at multiple angles. Using the distribution of the points to be measured on the standard sphere as a constraint condition, the model was optimized to obtain the actual pose and direction of the rotation axis and the relationship between the X-axis and the Z-axis. The optimization constraint function is shown in Equation (10), where *C_r_* is the center of the standard sphere, and *R* is the radius of the standard ball.

(10) $$f(x)=\min(\sum_{i}^{n}(\left|\left(P_{i}-C_{r}\right)\right|-R))$$

## 5. Experiment and Conclusions

### 5.1. Experiment System Calibration

**Figure 8:**
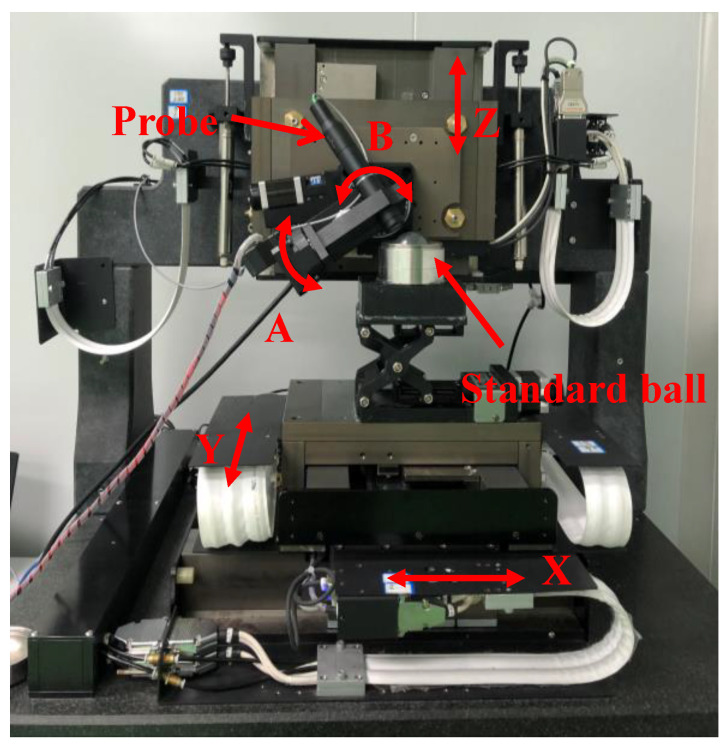
A photograph of the measurement system.

The experimental system consisted of a motion control part and a data measurement part. The motion control part consisted of five motion guides (X, Y, Z, B, A) and their associated control parts, as shown in Figure 8. The data measurement part consisted of a chromatic confocal sensor. The hardware parameters of each part are shown in Table 3. The calibration experiment was carried out by using a Taylor standard ball with the help of the calibration model presented in Section 3.

**Figure 9:**
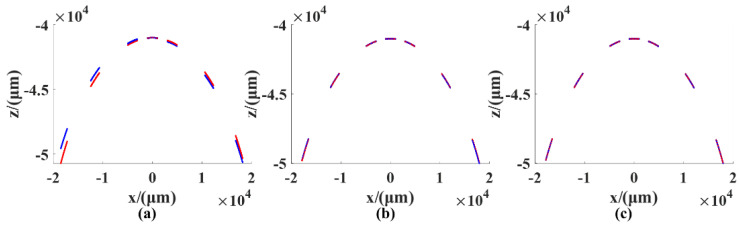
The measurement data’s distribution: (**a**) Before calibration; (**b**) after the calibration of the pose of the rotation axis; (**c**) after the calibration of the pose of the rotation axis and the XZ perpendicularity.

We use the calibration of the rotation axis B as an example. The rotation angles of 0°, ±10°, ±30°, and ±50° were selected for the measurement. The measurement data are shown in Figure 9, in which the blue color denotes measurement data and the red color denotes ideal data. Before the calibration, there was a large difference between the measurement data and the ideal data. After the pose of the rotation axis and the XZ perpendicularity were calibrated, the measured data basically coincided with the ideal data. The results of the calibration of the system are shown in Table 4.

**Figure 10:**
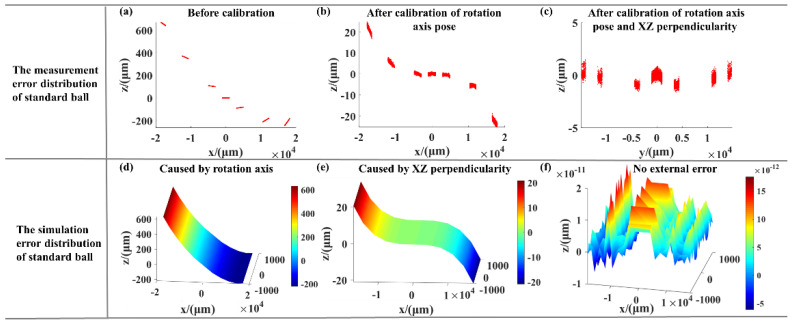
The distribution of error in the measurement data: (**a**) Before calibration of the pose of the rotation axis and the XZ perpendicularity; (**b**) After the pose of the rotation axis were calibrated; (**c**) After the pose of the rotation axis and the XZ perpendicularity were calibrated; The distribution of error in the simulation data: (**d**) Before calibration of the pose of the rotation axis and the XZ perpendicularity; (**e**) After the pose of the rotation axis were calibrated; (**f**) After the pose of the rotation axis and the XZ perpendicularity were calibrated.

To verify the correctness of the error model, the real pose of rotation axis B and XZ perpendicularity obtained by the experiment and shown in Table 4 were substituted into the error model as simulation values, and the simulation results were compared with the real measurement results. The measurement system calibration error PV of the standard ball before calibration of the pose of the rotation axis and the XZ perpendicularity was 932.788 μm (Figure 10a). After the pose of the rotation axis and the XZ perpendicularity were calibrated, the error PV was reduced to 67.225 μm (Figure 10b) and 3.112 μm (Figure 10c), respectively. The simulation error PV before calibration of the pose of the rotation axis and the XZ perpendicularity was 891.577 μm (Figure 10d). After the pose of the rotation axis and the XZ perpendicularity were calibrated, the simulation error PV was reduced to 53.587 μm (Figure 10e) and almost zero (Figure 10f), respectively. The distribution of the simulation error and the measurement error was basically consistent. As the actual calibration produced other errors, the error PV was not the same, but the order of magnitude was consistent, such as that for the dynamic error of the system. By comparing the actual measurement results with the simulation results, we were able to verify the correctness of the system simulation model and the calibration model.

### 5.2. Verification of System Calibration Results

**Figure 11:**
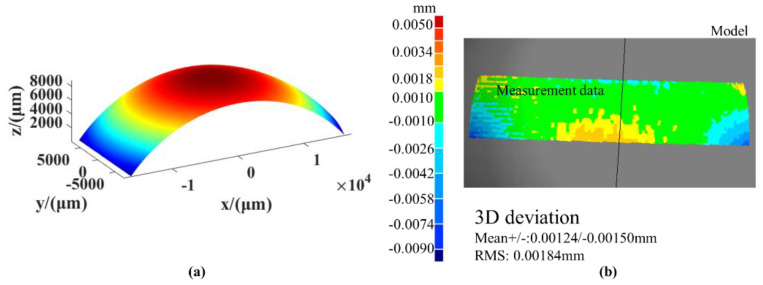
The measurement results: (**a**) The measured surface shape, (**b**) the measurement error.

Due to the angular tolerance of the probe, rotary scanning is required for workpieces with a high degree of curvature. However, before the system is calibrated, the position of the probe is unknown and the measurement range can easily be exceeded after the probe is rotated, resulting in no data signal from the probe at this position, which would make it impossible to measure the entire contour of the ball before calibration. After we performed the calibration experiment mentioned in Section 5.1, we obtained several key system error values and used the error model presented in Section 3 to compensate for them. Additionally, the measurement experiment with the standard ball was designed to verify the results of the calibration of the system. The scanning range of the standard ball was [–17.23 mm, 17.23 mm] in the X direction and [–7.5 mm, 7.5 mm] in the Y direction. The angle measured in the X direction was ±60° and the deflection angle measured in the Y direction was ±20° due to the limitations of the mechanical structure. The measurement results are shown in Figure 11. The final Root Mean Square (RMS) was 1.84 μm. As the dynamic error of the displacement table cannot be compensated for offline, some errors remain in the measurement of the standard ball. The standard ball was measured repeatedly to verify the repeatability of the measurement system. The results are shown in Table 5.

### 5.3. Measurement Application

**Figure 12:**
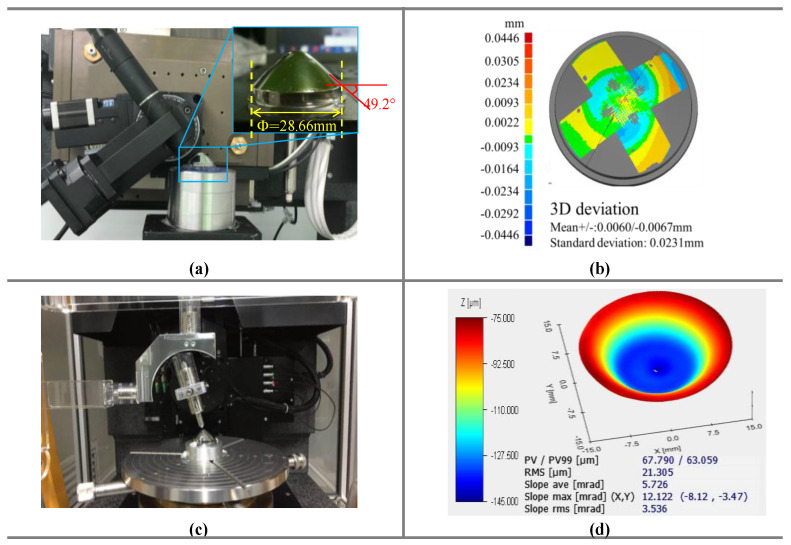
(**a**) The object measured by the system, (**b**) the measurement error of the system, (**c**) the object measured by LuphoScan, and (**d**) the measurement error of LuphoScan.

The aim of this study is to solve the problem of measuring a highly curved optical freeform surface. We proposed a rotary scanning measurement method, built the corresponding measurement system, and solved the error and calibration problems. Finally, we verified the feasibility of the proposed scheme by measuring two typical curved surfaces. First, we used the method proposed in this paper to measure a rotationally symmetric conical mirror. The maximum diameter (in the lower zone) of the conical mirror we measured was 28.66 mm, and its maximum inclination angle was 49.2°. Before measurement, the workpiece was scanned to unify the machining coordinate system and the measuring coordinate system. During measurement, the probe always followed the surface and was basically parallel to its normal direction. The measurement results are shown in Figure 12b. The RMS between the measured workpiece and the ideal surface was 23.1 μm. The results of repeated measurement of the workpiece are shown in Table 6.

To verify the accuracy of the measurement system, the commercial instrument LuphoScan was used to measure the conical mirror. The measurement results are shown in Figure 12d. From the results of the workpiece’s measurement with LuphoScan, the RMS was 21.305 μm. The measurement system obtained an average RMS of 24.224 μm after many experiments. The results show that there remained a certain deviation between the results obtained using the measurement system proposed in this paper and those obtained using the commercial instrument. The reason for the deviation is the dynamic error of the system that cannot be compensated for offline. Therefore, limited by the accuracy of the hardware used in the proposed system, the accuracy of the proposed measurement system is consistent with the actual measurement ability.

**Figure 13:**
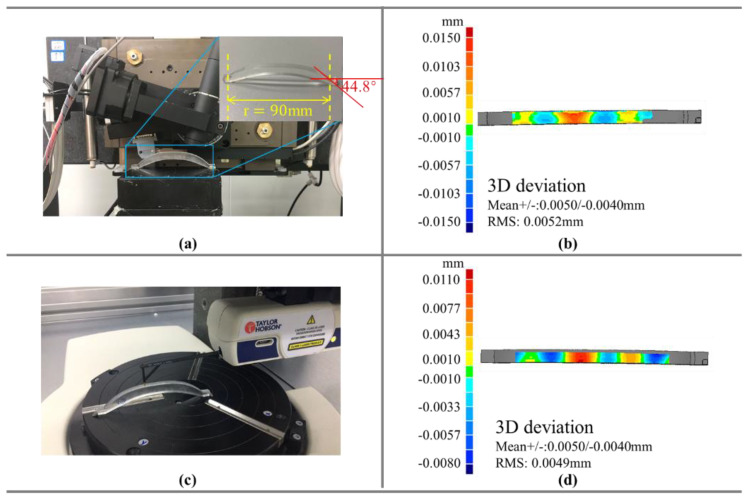
(**a**) The object measured by the system, (**b**) the measurement error of the system, (**c**) the object measured by a profilometer, and (**d**) the measurement error of the profilometer.

Traditional noncontact commercial instruments such as LuphoScan can only be used to measure rotationally symmetric workpieces. However, the measurement method proposed in this paper can be used to measure nonrotationally symmetric surfaces with a high degree of curvature. In this study, a nonrotationally symmetric F-theta mirror was measured to verify the feasibility of the system. The diameter of the conical mirror we measured was 90 mm, and the maximum inclination angle was 44.8°. However, because the two sides of the F-theta mirror were in an arc transition, the final measurement range was 70 mm after abandoning this part. The measurement results are shown in Figure 13b. The RMS was 5.2 μm between the measured workpiece and the ideal surface. The results of repeated measurement of the workpiece are shown in Table 7.

Because LuphoScan cannot measure nonrotationally symmetric surfaces, we used a contact profilometer to measure the F-theta mirror and compare the measurement results. The measurement results are shown in Figure 13d. From the results of the workpiece’s measurement with the profilometer, the RMS was 4.9 μm. The measurement system obtained the average RMS of 5.22 μm after many experiments.

## 6. Conclusions

In this paper, a new dual-axis rotational scanning measurement method was proposed for highly curved freeform surfaces, whose measurement is limited by the angular tolerance of the probe and the complexity of the system. The system error and calibration problems during actual measurement were solved, and the measurement of a highly curved freeform surface was finally realized. Our conclusions can be summarized as follows:

(1) An optical, noncontact, dual-axis rotary scanning measurement method was proposed to solve the problem of the traditional probe being limited by the measurement range and the angular tolerance. The optical probe can scan the surface profile while always keeping consistent with the normal vector of the measuring points with the help of the double rotation axis. This method can adapt to the changes in curvature in any direction on a highly curved freeform surface.
(2) We provided a system error calibration technique for rotation axis errors. The key system errors that affect the measurement accuracy were analyzed through the establishment of an error model. Then, the real error values were obtained by the optimal calculation in the calibration process and compensated for in the measurement process. The advantages of this method are that it avoids the additional use of high-precision equipment and calibrates the system’s accuracy conveniently and at low cost.
(3) We measured two highly curved freeform surfaces (a cone mirror and an F-theta mirror) in experiments. The experimental results show that the measurement accuracy of the system reaches the micron level. The feasibility of the measurement method was verified by comparing the results obtained using the proposed method to those obtained using commercial equipment.
(4) The proposed method can also be applied to correct the error in measurements obtained using other multi-axis measurement systems. It is worth noting that, due to the accuracy of the used hardware, the measurement accuracy of the system is limited. If hardware with higher accuracy is used in the future, the measurement accuracy will be further improved.

## Figures and Tables

**Table 1:** The error of the measurement system.

	Axis	Error
Dynamic Error	X-axis	*δ_xx_*, *δ_xy_*, *δ_xz_*, *θ_xx_*, *θ_xy_*, *θ_xz_*
	Y-axis	*δ_yx_*_,_*δ_yy_*, *δ_yz_*, *θ_yx_*, *θ_yy_*, *θ_yz_*
	Z-axis	*δ_zx_*, *δ_zy_*, *δ_zz_*, *θ_zx_*, *θ_zy_*, *θ_zz_*
	A-axis	*δ_ax_*, *δ_ay_*, *δ_az_*, *θ_ax_*, *θ_ay_*, *θ_az_*
	B-axis	*δ_bx_*, *δ_by_*, *δ_bz_*, *θ_bx_*, *θ_by_*, *θ_bz_*
Static Error	Perpendicularity Error	*α_zx_*, *α_zy_*, *α_xy_*
	Installation error of the A-axis	*T_ax_*, *T_ay_*, *T_az_*, *β_ax_*, *β_ay_*, *β_az_*
	Installation error of the B-axis	*T_bx_*, *T_by_*, *T_bz_*, *β_bx_*, *β_by_*, *β_bz_*

**Table 2:** The proportion of measurement system errors.

Number	Error	Surface Error PV (μm)	Proportion (%)
1	*θ_xx_*	0.695	0.038
2	*θ_xy_*	3.379	0.185
3	*θ_xz_*	1.511	0.083
4	*θ_yx_*	1.215	0.066
5	*θ_yy_*	3.379	0.185
6	*θ_yz_*	0.990	0.054
7	*α_zx_*	**22.057**	**1.212**
8	*α_zy_*	0.986	0.054
9	*α_xy_*	0.981	0.053
10	*T_y_*	0.089	0.005
11	*T_z_*	0.342	0.018
12	*f*(*B*_0_)	**891.577**	**49.021**
13	*f*(*A*_0_)	**891.577**	**49.021**

**Table 3:** The hardware parameters of the measurement system.

Hardware	Travel/Range	Accuracy	Others
X/Y/Z axes	200 mm	200 nm	\
B/A axes	360°	0.004°	\
Probe	400 μm	0.08 μm	NA: 28°
Standard ball	R = 22.4877 mm	PV: 38 nm	\

**Table 4:** The results of the calibration of the measurement system.

Error Term	Pose of the Rotation Axis	Direction of the Rotation Axis
Installation error of the B-axis	(546.8961, 1060.0933, 595.2549)	(0.0001, −0.9999, 0.0032)
Installation error of the A-axis	(−12.0467, −895.2491, 778.3344)	(0.9999, 0.0058, −0.0088)
XZ perpendicularity error	−0.07°	

**Table 5:** The results of repeated measurement of the standard ball.

Measurement Number	RMS (μm)
1	1.84
2	1.96
3	1.73
4	1.88
5	1.91
Mean	1.86

**Table 6:** The results of repeated measurement of the conical mirror.

Measurement Number	RMS (μm)
1	22.721
2	23.163
3	25.796
4	24.224
5	25.372
Mean	24.255

**Table 7:** The results of repeated measurement of the F-theta mirror.

Measurement Number	RMS (μm)
1	5.2
2	5.1
3	5.2
4	5.3
5	5.3
Mean	5.22
